# Development and Validation of a Novel General Medication Adherence Scale (GMAS) for Chronic Illness Patients in Pakistan

**DOI:** 10.3389/fphar.2018.01124

**Published:** 2018-10-09

**Authors:** Atta Abbas Naqvi, Mohamed Azmi Hassali, Mehwish Rizvi, Ale Zehra, Wajiha Iffat, Abdul Haseeb, Shazia Jamshed

**Affiliations:** ^1^Discipline of Social and Administrative Pharmacy, School of Pharmaceutical Sciences, Universiti Sains Malaysia (USM), Penang, Malaysia; ^2^DOW College of Pharmacy, DOW University of Health Sciences, Karachi, Pakistan; ^3^Department of Clinical Pharmacy, College of Pharmacy, Umm Al Qura University, Makkah, Saudi Arabia; ^4^Department of Pharmacy Practice, Kulliyah of Pharmacy, International Islamic University Malaysia, Kuantan, Malaysia

**Keywords:** medication adherence, patient compliance, medication persistence, chronic illness, patients, Pakistan

## Abstract

**Objective:** This study aimed to develop and validate a self-reporting adherence tool termed as General Medication Adherence Scale (GMAS) in Urdu language for measuring adherence toward medication use among Pakistani patients with a chronic disease.

**Methods:** A month-long study (December 2017) was conducted in three tertiary health care settings of Karachi, Pakistan. The tool underwent content and face validity as well as factor analyses, i.e., exploratory, partial confirmatory and confirmatory factor analyses. Random sampling was conducted, and sample size was calculated using item response theory. The item-to-respondent ratio was 1:15. Fit indices namely normed fit index (NFI), Tucker Lewis index (TLI), comparative fit index (CFI), goodness of fit index (GFI), absolute goodness of fit (AGFI), parsimony goodness of fit index (PGFI), root mean square error of approximation (RMSEA), and standard root mean square residual (SRMR) were calculated. Additionally, estimation of the convergent, discriminant and known group validities, was conducted. Internal consistency was analyzed by test-retest reliability, McDonald’s and Pearson correlation coefficient. The factor analyses were conducted using IBM SPSS version 22 and IBM SPSS AMOS version 25.

**Results:** Content validity index (CVI) was reported at 0.8 (SD 0.147) and the tool was content validated with three hypothetical constructs. Factor analyses highlighted a 3-factor structure. The fit indices were calculated with satisfactory results, i.e., PGFI, GFI, AGFI, NFI, TLI, and CFI were greater than 0.9 and PGFI > 0.5. The values of RMSEA and SRMR were less than 0.07. A Cronbach’s alpha value of 0.84 was obtained in reliability analysis. The test-retest Pearson’s correlation coefficient value was reported at 0.996 (*p*-value < 0.01). Convergent and discriminant validities for all constructs and, known group validity for two constructs, were established. A high response rate of 91% was achieved in respondents. Patients without insurance coverage appeared to be low adherent compared to those with insurance coverage (*p*-value < 0.05). Non-comorbid patients were more likely to be highly adherent as compared to comorbid patients (*p*-value < 0.01).

**Conclusion:** A novel tool GMAS was developed in Urdu language and was subsequently validated in patients with chronic diseases.

## Introduction

Patient adherence to medications is a challenging issue faced by the healthcare providers around the globe. According to World Health Organization (WHO), non-adherence is measured by relating patients’ medication taking behavior with the prescribed regimen. Patient medication adherence may be defined as adherence to medications for an illness as prescribed (Sabate, 2003; Vrijens et al., 2012).

Non-adherence to medication therapy may increase the likelihood of suffering from adverse treatment outcomes. This could result in increased hospital admissions and financial costs that are incurred on patients as well as the healthcare system (Iuga and McGuire, 2014). Studies report that around 10% of hospitalizations in United States are consequence of non-adherence (Vermiere et al., 2001; Sokol et al., 2005). Around 3–10% of avoidable healthcare cost in United States has been reported to be linked to non-adherence (Benjamin, 2012; Iuga and McGuire, 2014). This cost varies with diseases, patient traits and insurance coverage. It may reduce a patient’s quality of life as well as increase morbidity and mortality (DiMatteo, 2004; Vrijens et al., 2008). Patients of chronic illnesses require continuous medication therapy to manage their disease state. Chronic diseases are prolonged illnesses that cannot be completely cured and require long term medication therapy (Sabate, 2003). Adherence to medication regimen is one of the ways to ensure better chronic disease state management and improved treatment outcomes (Martin et al., 2005). Measuring adherence to medications is essential to monitor treatment goals and understand patient psyche. This may be affected by patient behavior in response to factors such as comorbidity, additional pill burden, regime complexity and financial barriers (Martin et al., 2005; Viana et al., 2014). It may be difficult to predict these factors with conventional techniques.

Measuring patient medication adherence can be achieved by several direct and indirect methods. Direct methods include patient observation by a healthcare provider or, measuring serum drug levels in laboratory. Indirect methods involve inquiring the pharmacy about dispensed medications for a patient however, this would not be indicative of patient’s medication taking behavior at home. For instance, patients may forget or skip a dose amid additional pill burden but refill prescription at the stated date. A relatively easy and inexpensive indirect method to measure patient medication adherence involves use of validated questionnaires (Iuga and McGuire, 2014). Measuring adherence may help in identifying patient needs and requirement of advice or counseling by practitioner to improve patient’s decision-making ability toward adhering to medication regimen and foster treatment satisfaction (Martin et al., 2005).

Several psychometric tools have been formulated to measure non-adherence to medications in patients; however, no tool has originated from developing countries. Some medication adherence reporting instrument namely the Morisky’s medication adherence scale developed by Morisky and colleagues, the modified drug adherence workup tool (MDRAW) as well as Adherence to refills and medications scale by Kripalani and colleagues are available (Morisky et al., 1986; Kripalani et al., 2009; Lee et al., 2017). However, the important issue of cost related non-adherence (CRNA) is often neglected. Though, the latter two have incorporated this aspect in a single item in the questionnaire that asks patients if cost proves to be too much to buy a medicine. However, they do not measure CRNA as a single domain, i.e., if the patient feels the medicines are unworthy of costs paid. Patients may tend to forego their treatment in face of a financial constraint or socio-economic burden. This may become a barrier to treatment and eventually result in CRNA (Kurlander et al., 2009). Studies have reported high drug costs and non-coverage of medicines as determinant of non-adherence (Kennedy et al., 2008; Iuga and McGuire, 2014; Zhang et al., 2014). Though, existing medication adherence measurement tools have incorporated different aspects of patient perception in measuring adherence however, non-adherence to medication therapy due to inability to pay direct cost has not been adequately addressed in any instrument. The out-of-pocket expenditure is an important area of health economics specially in developing countries where most patients pay direct medical costs (Hussain et al., 2014; Naqvi et al., 2016). Thus, the existing tools lack this important domain in measurement of adherence.

This highlights a need to develop a medication adherence measuring instrument that incorporates all pre-existing domains of patient psycho-behavioral aspects including CRNA. There is a dearth of knowledge regarding medication adherence in Pakistani patients. A qualitative study by Naqvi and colleagues highlighted patient perceived barriers to medication adherence (Naqvi et al., in press). The findings of Naqvi et al. (in press) helped us in development of a tool to measure medication adherence in this population. The purpose of this study was to develop and validate a novel research instrument known as the General Medication Adherence Scale (GMAS) in Urdu language to measure medication adherence in Pakistani patients with chronic diseases.

## Methods

A month-long study (December 2017) was conducted in three tertiary healthcare settings of Karachi, Pakistan.

### Patient Recruitment and Venue of Study

The study was conducted in out-patient departments (OPDs) and pharmacies of tertiary care hospitals on weekdays (Monday–Saturday) during evening hours (6–9 pm). Patients visited the clinics in these timings.

Patients were randomized based on their medical record number (MRN). Every chronic illness patient visiting OPD and out-patient pharmacy, with a MRN ending with an odd number, was invited to participate in the study. In the OPDs, patients were checked for a chronic condition by observing their medical history. Secondly, patients visiting out-patient pharmacy were also checked for any chronic illness by their prescription, i.e., if it had medication/s prescribed for chronic illnesses.

### Participants and Eligibility Criteria

The participants in our study were patients of Pakistani origin suffering from chronic illnesses. Male and female patients (above 18 years) with a chronic illness diagnosed at least 3 months ago and, with or without comorbidities were invited to be a part of the study. Those who agreed to participate were included in the study and were handed the questionnaire. Patients with hearing and vision disability who were accompanied by a caregiver were also encouraged to participate. Non-Pakistanis, in-patients, acutely ill patients as well as non-consenting patients were not included in the study.

## Phases of Development

### Conceptualization of Research Instrument

The questionnaire was designed as a self-report tool which was filled by patients or patients’ caregiver in case the patient had vision or hearing disability. All questions were in Urdu language and multiple-choice format; patients had to select one answer that best reflected their medication taking behavior. A male and a female research assistant was available to facilitate in case patients had any difficulty.

The sample size was calculated from a statistical aspect. Several studies have suggested sample size concerning factor analyses. Osborne JW and Costello AB suggested large sample size and/or an item-to-respondent ratio of at least 1:5 up to 1:10 (Osborne and Costello, 2004; Dowrick et al., 2005). Since the GMAS had 11 items, the ideal sample size was thought to be around 55 to 110 patients. However, the study gathered response from 161 patients resulting in an item to respondent ratio of 1:15, i.e., more than the minimum required sample count.

The first phase of the study was the generation of items for the development of the GMAS. The tool was developed in Urdu language. It was conceived from 2 major concepts. The concept of adherence to medication as described by taxonomy of Vrijens et al., was considered in designing constructs for GMAS, i.e., patient behavior related non-adherence as well as additional disease and pill burden related non-adherence. This concept is defined as a process by which patient’s medicine taking behavior corresponds to the prescribed therapeutic regimen (Vrijens et al., 2012). Secondly, unaffordability of medications or out-of-pocket expenditure on medications was also considered as potential construct for our research instrument. Pakistan spends a mere United States Dollar (USD) 14 per capita on its health care structure which is below the WHO recommended USD 34. Zaidi et al. (2013) highlighted the financial barriers to treatment and affordability of medicines as policy concerns for Pakistan as mean out-of-pocket expenditure ranged from PKR 198–252 per prescription. The affordability index per month for a chronic patient’s treatment in Pakistan is more than a day’s income of a lowest paid public-sector employee. Furthermore, cost of medication therapy for chronic illness was observed to be unaffordable even with generic medications (Zaidi et al., 2013). Studies conducted in Pakistan have reported that the out-of-pocket expenditures are regarded as treatment barriers by patients and their healthcare providers (Hussain et al., 2014; Naqvi et al., 2016). Thus, it is important to document medication taking behavior of patients of developing countries like Pakistan as financial barriers have potential to decrease adherence to prescribed regimen.

### Tool Elaboration

The initial draft of the tool was prepared by a clinical pharmacist and university professor in consultation with medical practitioner. The process of development started with generation of items. It involved a literature review of existing research instruments used for measuring medication adherence. The working group finalized 11 questions in three distinctive constructs. Each question had four possible outcomes. Our tool measured non-adherence to medications and not the quantity of doses missed due to non-adherence.

The standardization of GMAS included face and content validity followed by a pilot study.

### Expert Panel

A panel of experts was set up for the face and content validation of the research instrument. The panel consisted of four university professors, a clinical pharmacist, four community pharmacists, six medical practitioners, and a social scientist. University professors helped with face and content validation procedures, pharmacists and medical practitioners provided their insight in to understanding of items in the tool. A social scientist provided a view of patient’s perceptions regarding adherence. The aim was to address three concerns; relevance and clarity of drafted questions, applicability of GMAS to chronic patients and feasibility of research tool in a global context. This process ensured face and content validity of the tool.

### Content and Face Validity

Content and face validity were assessed by handing the research instrument to 19 respondents who were; pharmacists working in community pharmacies and hospitals, practitioners, university professors, as well as a social scientist. Content validity for each item as well as content validity index (CVI) was calculated (Lawshe, 1975; Rungtusanatham, 1998). Significant items were retained whereas non-significant items were excluded. The face and content validity were established at this point.

### Pilot Testing for Clarity and Acceptability of GMAS

**FIGURE 1:**
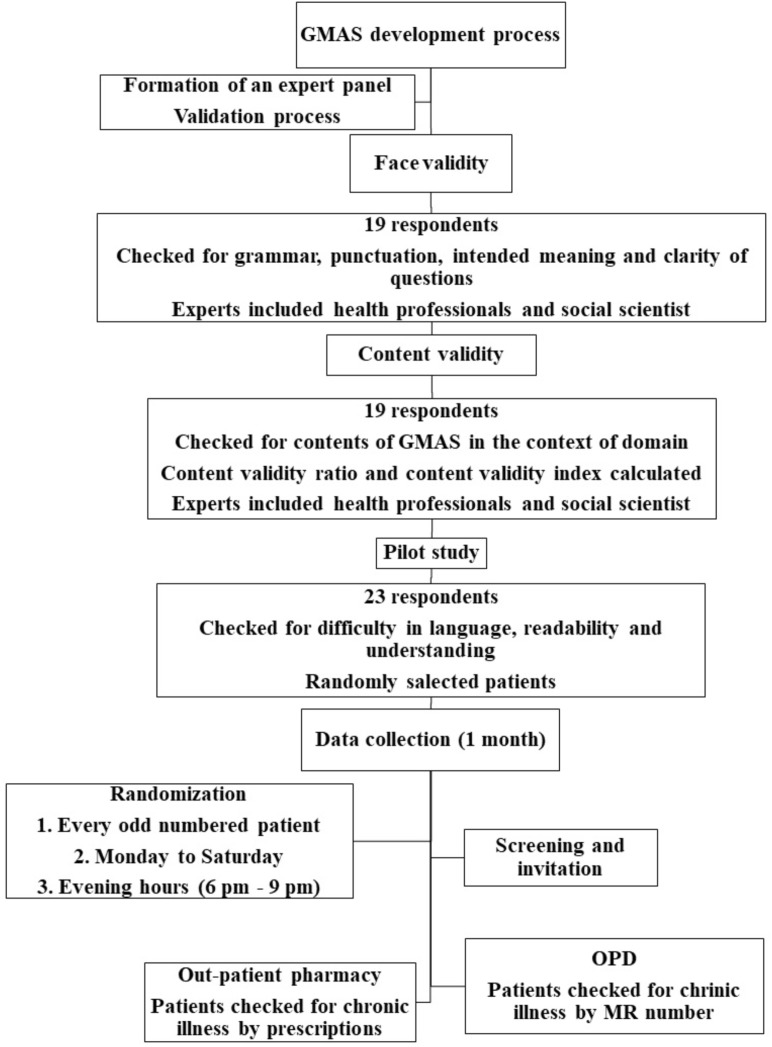
GMAS concept, design and standardization.

A multi-step pilot testing was conducted. At first, it was handed to 23 random patients to check for any difficulty in language, understanding and construct. Neither any difficulty in language and construct was observed, nor any difference between intention and understanding of question was noted. At this point the tool was deemed fit to use in the population (**Figure 1**).

### Factor Analyses and Evaluation of Model Fit

The second step included administering the questionnaire in the sample. This was done with item-to-respondent ratio of 1:10 to explore factor structure (Williams and Brown, 2010). The questionnaire was administered to a larger sample size with a ratio of 1:15 in third step to confirm factor structure with fit indices. Absolute fit indices namely goodness of fit (GFI), absolute goodness of fit (AGFI), root mean square error of approximation (RMSEA) and standardized root mean square residual (SRMR) was calculated. These indices highlight a good model fit (Jöreskog and Long, 1993). Additionally, incremental fit indices such as normed fit index (NFI), Tucker Lewis index (TLI), comparative fit index (CFI) were also noted. A value of GFI, AGFI, and NFI was > 0.90 and for TLI, CFI > 0.95, RMSEA, SRMR values < 0.07, indicate good model fit (Pett et al., 2003; Hair et al., 2009; Shima et al., 2015). A value for parsimony goodness of fit index (PGFI) > 0.5 was considered satisfactory (Mulaik et al., 1989). Structure equation modeling was carried out using IBM SPSS AMOS version 25.

### Levels of Patient Medication Adherence

Based on literature review, we categorized 5 levels of patient medication adherence namely, high, good, partial, low and, poor adherence. A patient is categorized as having; high adherence if the score is > 90%, good adherence if the score is between 71 and 89%, partial adherence if the score is between 50 and 70%, low adherence if the score obtained is greater than 30% but less than 50% and, poor adherence if it is below 30%.

### Scoring Criteria

The GMAS measured adherence across three domains namely non-adherence due to patient behavior (PBNA), i.e., un-intentional and intentional non-adherence, comorbidity and pill burden related non-adherence (ADPB) and, cost-related non-adherence (CRNA). The adherence to medication is measured for each domain individually and cumulatively as well. Patients answer 11 questions and are graded out of a score of 33. Grading for cumulative medication adherence was described as; score between 30 and 33 was considered as high adherence, good adherence was considered for a score between 27 and 29, partial adherence was considered if final score is between 17 and 26, low adherence was considered for patients having a score between 11 and 16. Patients whose final score was between 0 and 10 would be classified as poorly adherent.

For individual domains, a total of 5 items were available for patient behavior related non-adherence followed by 4 items in additional disease and pill burden domain. CRNA comprised of 2 items. All items had 4 possible outcomes, i.e., always, mostly, sometimes and never, that awarded scores 0, 1, 2, and 3, respectively.

The score for GMAS 1 domain was calculated from 15. The scoring criteria was described as high adherence for a score between 13 and 15, good adherence for a score between 11 and 12, partial adherence for a score between 8 and 10, low adherence for a score between 5 and 7 and poor adherence for a score less than or equal to 4. Similarly, maximum score for GMAS 2 domain was 12. High adherence was designated for patient with a score between 11 and 12, good adherence was considered for a score between 9 and 10, partial adherence was designated for a patient with a score between 6 and 8. Patients with a score between 4 and 5 were categorized as low adherent and score below 4 highlighted poor adherence. A maximum score of 6 could be achieved for the GMAS 3 domain. A patient exhibited; high adherence if score was 6, good adherence if the score was 5, partial adherence if the score was between 3 and 4, low adherence if the score was 2 and poor adherence if it was below 2.

### Evaluation of GMAS Adherence Measurement

The measurement property of GMAS was evaluated by reviewing validity and reliability. Convergent validity was assessed by calculating average factor loading of a construct. The validity for a construct was established if the average factor loading was greater than 0.7. Discriminant validity was also assessed by calculating the average variance and correlation coefficient between two constructs. Discriminant validity for a construct was established if average variance was greater than squared correlation coefficient. Internal consistency was measured by test-retest method using Cronbach’s alpha (α) values. A value of 0.5 or greater was considered acceptable. Furthermore, item-to-total correlation (ITC) was calculated and a value greater than 0.2 was considered acceptable (Streiner and Norman, 1995; Bowling, 2009; Sushil and Verma, 2010). Pearson’s correlation coefficient (ρ) was used to assess the test-retest reliability between two time-points with a gap of 4 weeks. A value of (ρ) more than 0.75 and *p*-value < 0.05 was considered significantly strong correlation (Lahey et al., 1983; Cohen, 1988; De Vellis, 1991). Inter-rater reliability, McDonald’s coefficient and intra-class correlation (ICC) was also calculated (Lahey et al., 1983; Cohen, 1988).

### Known Group Validity

The study hypothesized that known patient groups such as those with or without comorbidity and health insurance coverage were more likely to appear low adherent in respective constructs (Kurlander et al., 2009; Iuga and McGuire, 2014). This was evaluated by cross tabulation and Fisher’s Exact test for association. A *p*-value of less than 0.05, was considered significant.

### Sensitivity Analysis

Sensitivity of GMAS was evaluated to screen patients with low adherence due to polypharmacy and out-of-pocket expenditure.

### Ethical Approval and Consent

This research paper is based on a research project approved by Institutional Review Board of Allied Med Ethics (Reference number: NOV:15), and Clifton Central Hospital (Letter#24082017-2), Karachi, Pakistan. An informed consent was obtained from respondents before data collection.

## Results

### Scale Construct and Items Generation

A total of 5 domains were identified. The initial item pool consisted of 20 questions. This draft underwent face and content validity and was subsequently modified with 3 constructs and 11 items (**Supplementary Figure S1**).

### Face and Content Validity

Based on the expert panel feedback, first item of the GMAS 1 construct was removed. Two constructs were merged together to form a single construct, i.e., GMAS 1. One construct was removed, and some items were modified for clarity. After face validity, content validity of tool was also assessed by respondents. The CVI was reported at 0.8 (SD 0.147) and content validity ratios (CVR) are reported in **Table 1**.

**Table 1:** Content validity ratio (CVR) and factor structure.

Construct/Subscale	Items	CVR	Factors		
			**1**	**2**	**3**
GMAS 1 (PBNA)	1	0.99	0.592	–	–
	2	0.87	0.798	–	–
	3	0.87	0.722	–	–
	4	0.62	0.846	–	–
	5	0.75	0.803	–	–
GMAS 2 (ADPB)	6	0.99	–	0.734	–
	7	0.99	–	0.846	–
	8	0.5	–	0.930	–
	9	0.87	–	0.558	–
GMAS 3 (CRNA)	10	0.75	–	–	0.972
	11	0.62	–	–	0.504


*PBNA, Patient behavior related non-adherence; ADPB, Additional disease and pill burden; CRNA, Cost related non-adherence; CVR, Content validity ratio.*

### Piloting of Instrument in Patients

The instrument was handed to 177 patients out of which 161 patients provided their information. The response rate achieved was 91%. A total of 96 patients (59.6%) were male patients. The average age of patients was reported at 54 years (*X* = 54.1 ± 1.14). The demographic information is mentioned in (**Table 2**).

**Table 2:** Demographic information of patients.

	Patient information	Sample (*N*)	Percentage (%)
1.	**Gender**		
	Male	96	59.6
	Female	65	40.4
2.	**Marital status**		
	Married	138	85.7
	Single	23	14.3
3.	**Educational status**		
	Secondary	4	2.5
	Matriculate	9	5.6
	Intermediate	9	5.6
	Graduate	82	50.9
	Post graduate	19	11.8
	No formal education	38	23.6
4.	**Occupation**		
	Household	53	32.9
	Employed	42	26.1
	Retired	37	23
	Self-employed	20	12.4
	Un-employed	9	5.6
5.	**Monthly family income**		
	Less than PKR 10,000 (< US $ 81.14)	3	1.9
	Between PKR 10,000 and PKR 25,000 (US $ 81.14–202.84)	11	6.8
	Between PKR 25,000 and PKR 50, 000 (US $ 202.84–405.68)	61	37.9
	More than PKR 50,000 (US $ 405.68)	86	53.4
6.	**Ethnicity**		
	Urdu speaker	77	47.8
	Punjabi	53	32.9
	Pashtun	13	8.1
	Sindhi	10	6.2
	Baloch	8	5
7.	**Residence**		
	Urban	139	86.3
	Rural	22	13.7
8.	**Duration of illness**		
	Less than 1 year	3	1.9
	More than 1 year but less than 3 years	13	8.1
	More than 3 years but less than 5 years	51	31.7
	More than 5 years but less than 10 years	71	44.1
	More than 10 years	23	14.3
9.	**Type of chronic illness**		
	Endocrine and metabolic disorders (DM, thyroid, Grave’s disease, obesity)	28	17.4
	Cardiovascular and cerebrovascular disorders (HTN, CAD, CHF, Stroke)	54	33.5
	Pulmonary disorders (Chronic bronchitis, asthma, COPD, Emphysema)	24	14.9
	Musculoskeletal disorders (RA, OA, Osteoporosis, Gout)	9	5.6
	Gastrointestinal and liver disorders (IBS, IBD, hepatitis)	16	9.9
	CNS disorders (Epilepsy, Alzheimer’s disease, bipolar disease, Parkinson’s disease)	14	8.7
	Genetic and auto-immune disorders (Myasthenia gravis, cystic fibrosis, multiple sclerosis, Huntington’s disease)	7	4.3
	Kidney and prostate related disorders (CKD, BPH)	9	5.6


*HTN, hypertension; CAD, Coronary artery disease; CHF, congestive heart failure; COPD, chronic obstructive pulmonary disorder; RA, rheumatoid arthritis; OA, osteoarthritis; IBS, irritable bowel syndrome; IBD, inflammatory bowel disease; CKD, chronic kidney disease; BPH, benign prostate hyperplasia. The value of United States dollar ($) corresponds to exchange of Pakistani Rupee (PKR) to US $ at the time of this writing, i.e., 29th August 2018.*

### Factor Analyses and Model Fit Evaluation

**FIGURE 2:**
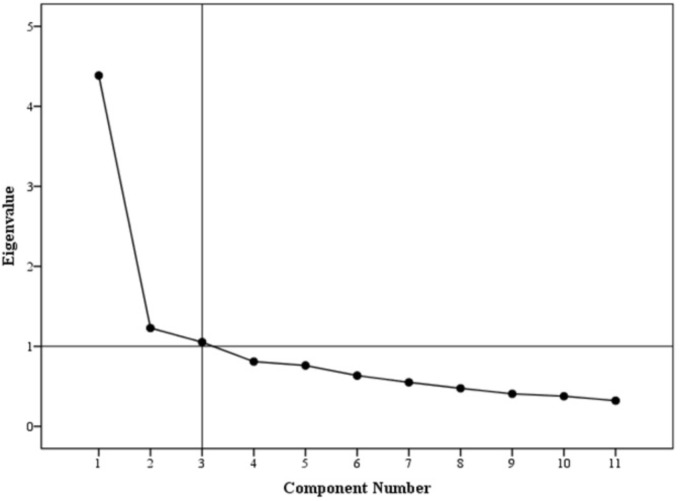
Scree plot.

Exploratory factor analysis (EFA) was conducted. Principle component analysis with Promax rotation was employed to analyse factor structure. The Kaiser-Meyer-Olkin (KMO) measure of sampling adequacy was reported at 0.8 with significant result for Bartlett’s test of sphericity, i.e., *p*-value < 0.001. A 3-factor model solution was obtained with eigenvalues above 1.0, accounting for 59.6% of variance (**Figure 2**). For demonstration of a clear model structure, items with factor loadings greater than 0.4 on a component, and non-salient loading less than 0.4 on other components, were considered as a single factor. Factor 1 contained 5 items, factor 2 contained 4 items and factor 3 contained 2 items. Two items per factor is a necessary pre-requisite in principle component analysis (Zwick and Velicer, 1986; Toll et al., 2007).

Items loaded on factor one measured intentional and unintentional non-adherence. Items loaded on factor two tend to measure non-adherence of a patient due to additional disease and pill burden. Factor three had 2 items that measured CRNA (**Table 1**). This model was then confirmed in the second sample by conducting a partial confirmatory factor analysis (PCFA) using Maximum likelihood method with Direct oblimin rotation. The value for KMO was reported at 0.86 and Bartlett’s test was significant with *p*-value < 0.001.

**FIGURE 3:**
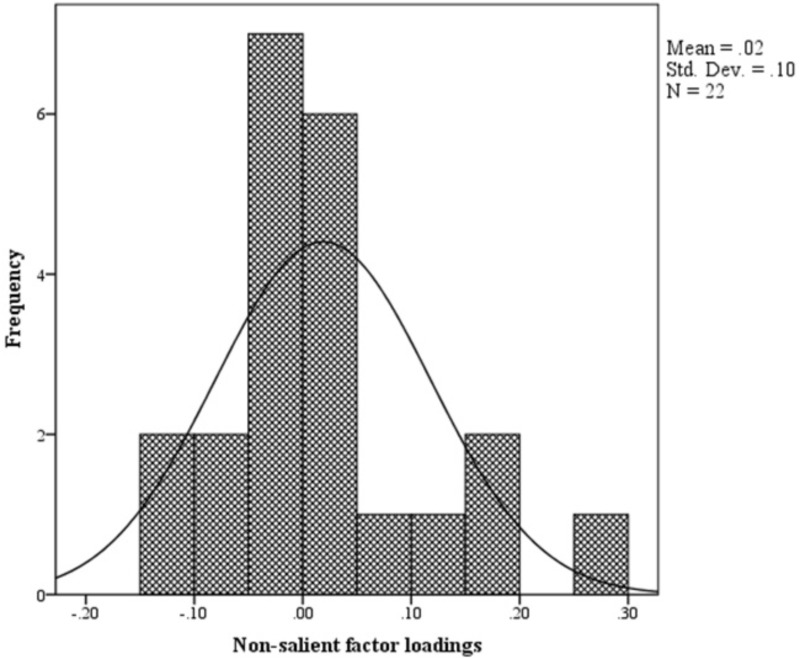
Histogram.

**FIGURE 4:**
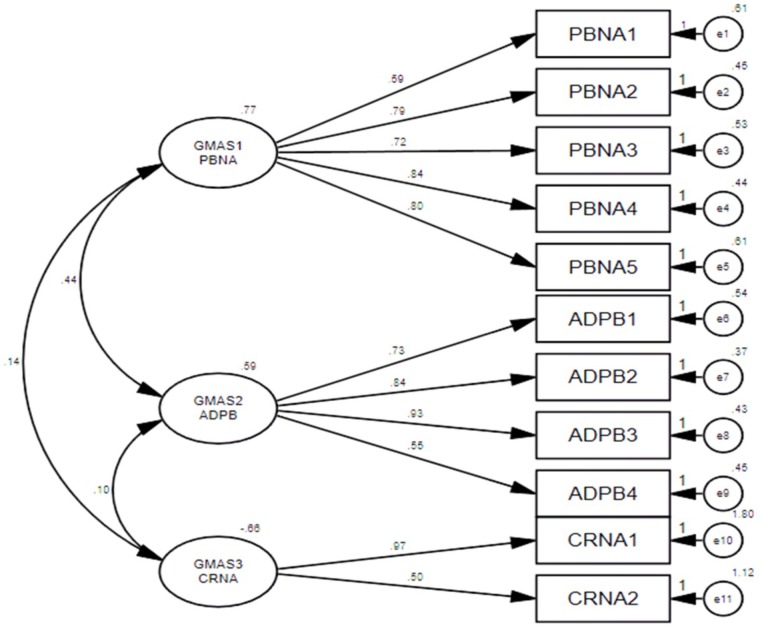
Structure equation model (SEM).

The distribution of non-salient factor loadings was normal with a mean of 0.2 (**Figure 3**). The null-model chi square obtained was 1443.147 (*df* = 55) and implied model chi square value was reported at 74.695 (*df* = 25). Fit indices were calculated using these values. The values obtained for fit indices in PCFA were; NFI = 0.95, TLI = 0.92, and CFI = 0.96, i.e., > 0.9. The value for RMSEA and SRMR was 0.06 and 0.03, respectively, i.e., less than 0.07. All these values confirmed a good 3-factor model fit. Further to this, a confirmatory factor analysis was conducted using IBM SPSS AMOS version 25. Structure equation modeling was conducted (**Figure 4**) and all previous fit indices were calculated including the GFI, AGFI, and PGFI. The fit indices were as follows; NFI = 0.92, TLI = 0.95, and CFI = 0.96. The value for RMSEA was 0.05. The values for GFI, AGFI, and PGFI were 0.95, 0.93, and 0.59, respectively (**Figure 4**).

### Convergent and Discriminant Validity

The average factor loadings for each construct was calculated. All 3 constructs of GMAS reported average factor loadings greater than 0.7, i.e., GMAS 1 = 0.752, GMAS 2 = 0.742, and GMAS 3 = 0.738, that established their convergent validity. Average variance between the constructs and squared correlation coefficient (r^2^) was calculated. The average variance between GMAS 1 and GMAS 2 was reported at 0.58 and *r^2^* = 0.26. Similarly, average variance between GMAS 1 and GMAS 3 was 0.59 and r^2^ was reported at 0.1. Finally, the average variance between GMAS 2 and GMAS 3 was 0.62 and r^2^ was reported at 0.09. The average variance values among the three constructs were greater than their respective r^2^ values. This established the discriminant validity among three constructs.

### Internal Consistency

The overall reliability of GMAS for 11 items was 0.84. All items were positive correlated with each other. The first construct reported (α) value of 0.806, all items demonstrated an item-total correlation (ITC) greater than 0.4. The second construct reported an α value of 0.778 with all items demonstrating a high ITC greater than 0.5. The third construct reported an α value of 0.445 with ITC greater than 0.2 (**Table 3**). The test-retest reliability was checked by correlating the adherence scores of participants at time-point 1 and 2. The test-retest Pearson’s correlation coefficient was 0.996 (*p*-value < 0.01) (**Table 3**).

**Table 3:** Internal consistency results of GMAS.

Construct/Subscale	Items	Variable	Corrected ITC	(α) if Item Deleted	R	*ω*	ICC	95% CI
GMAS 1 (PBNA)	5	V1	0.441	0.832	0.806	0.86	0.806	0.775–0.835
		V2	0.562	0.823				
		V3	0.610	0.818				
		V4	0.630	0.816				
		V5	0.598	0.819				
GMAS 2 (ADPB)	4	V1	0.602	0.819	0.778	0.9	0.778	0.741–0.811
		V2	0.556	0.823				
		V3	0.508	0.827				
		V4	0.512	0.827				
GMAS 3 (CRNA)	2	V1	0.226	0.852	0.445	0.75	0.445	0.326–0.542
		V2	0.483	0.829				
Overall	11				0.84			


*ITC, Item-total Correlation; α, Cronbach’s alpha; ICC, Intra-class correlation; R, Reliability; *ω*, McDonald’s coefficient.*

### Known Group Validity

The known group validity was observed by cross tabulating the adherence score with patient demographic information. Adherence scores of second construct, i.e., GMAS 2 (additional disease and pill burden) and, third, i.e., GMAS 3 (CRNA), were associated with comorbidity status and health insurance coverage, respectively. Non-comorbid patients with high adherence were observed in higher sample count as compared to comorbid patients with high adherence (*p*-value < 0.01). Most patients with no health insurance coverage had poor adherence as compared to those with full or partial insurance coverage (*p*-value < 0.05) (**Table 4**).

**Table 4:** Cross tabulation between patient groups and adherence scores.

Comorbidity	Poor adherence	Low adherence	Partial adherence	Good adherence	High adherence
**GMAS 2 (Additional disease and pill burden)**					
Yes	2^∗^ (3)^∗∗^	8^∗^ (5)^∗∗^	17^∗^ (16)^∗∗^	20^∗^ (16)^∗∗^	13^∗^ (20)^∗∗^
No	6^∗^ (5)^∗∗^	5^∗^ (8)^∗∗^	26^∗^ (27)^∗∗^	23^∗^ (27)^∗∗^	41^∗^ (34)^∗∗^

**Health insurance coverage**	**Poor adherence**	**Low adherence**	**Partial adherence**	**Good adherence**	**High adherence**

**GMAS 3 (Cost-related non-adherence)**					
Full coverage	1^∗^ (0)^∗∗^	1^∗^ (2)^∗∗^	12^∗^ (9)^∗∗^	0^∗^ (1)^∗∗^	1^∗^ (1)^∗∗^
Partial coverage	0^∗^ (2)^∗∗^	4^∗^ (6)^∗∗^	28^∗^ (24)^∗∗^	3^∗^ (4)^∗∗^	3^∗^ (3)^∗∗^
No coverage	7^∗^ (6)^∗∗^	21^∗^ (17)^∗∗^	60^∗^ (68)^∗∗^	13^∗^ (11)^∗∗^	7^∗^ (7)^∗∗^


*^∗^Observed count; ^∗∗^expected count.*

### Sensitivity Analysis

GMAS was sensitive (> 74%, *p*-value < 0.01) while screening patients with low adherence due to polypharmacy and out-of-pocket expenditure.

## Discussion

Documenting medication adherence by self-reporting tool is one of the most common, effective and less expensive way of assessing patients’ concordance to pharmacotherapy (Forbes et al., 2018). Several self-reporting adherence tools had been formulated previously. Those included the Shea scale (Dunbar-Jacob et al., 2012), Brief Medication Questionnaire (BMQ) (Nori et al., 2014), Adherence to Refills and Medications Scale (ARMS) (Nordstrom et al., 2013) and the Morisky’s Medication Adherence Scale (MMAS) (Shalansky et al., 2004) had been used before. However, studies have reported that no scale could be considered as a standard for measuring adherence (Forbes et al., 2018; Naqvi and Hassali, 2018). The MMAS and BMQ were reported to be too difficult for patients while Shea scale lacks adherence measurement based on patient behavior. MMAS scale is quite expensive to use as well. Besides, none of the scales measure CRNA (Forbes et al., 2018; Naqvi and Hassali, 2018). Since, all above mentioned scales originated in developed countries, absence of CRNA measurement is logical as most patients in those countries do not have to bear out-of-pocket medical expenditures.

We designed GMAS considering the inadequacies highlighted in previous instruments. The measurement purification was carried out by studying factor analyses. A three-factor structure was explored and confirmed in the sample that corresponded to the content validated version of the GMAS. Each item of a hypothetical construct loaded on a single component. Fit indices were calculated for the GMAS. The values for NFI, TLI, and CFI were greater than 0.9 and values obtained for SRMR and RMSEA were less than 0.07. All these values indicated a good model fit.

The evaluation of the measurement was determined by studying validity and reliability. The GMAS was subjected to convergent and discriminant validity. Convergent validity was evaluated by comparing the average factor loadings for each construct to the threshold value of 0.7. The factor loadings for all individual components were greater than 0.7 that established convergent validity. Discriminant validity was studied by comparing the average variance between the constructs and squared correlation coefficient. All constructs reported average variance greater than their respective squared correlation coefficients that established discriminant validity.

The internal consistency of the GMAS was also evaluated. A Cronbach’s alpha value of 0.84 was observed for 11 items of GMAS. This was higher than alpha value reported by MMAS-8 scale amongst hypertensive patients in Pakistan, i.e., 0.701 (Saleem et al., 2012; Abbas et al., 2015). Inter-rater reliability, composite reliability and intra-class correlations were also studied. All the items of the scale were positive correlated with each other with moderate to strong correlations. The Cronbach’s alpha values for individual constructs, i.e., GMAS 1 (PBNA), GMAS 2 (ADPB), and GMAS 3 (CRNA) were 0.8, 0.778, and 0.445, respectively. The ITC value for the three constructs were greater than 0.4, 0.5, and 0.2, respectively. The test-retest reliability was greater than 0.9, i.e., higher than the value reported by MMAS-Urdu (Saleem et al., 2012). The tool demonstrated a high acceptability among patients with a response rate of over 90% that exceeded the response rate achieved by MMAS scale in studies conducted among Pakistani patients (Saleem et al., 2012; Abbas et al., 2015).

This study established several strengths of GMAS, i.e., availability in Urdu language, better patient acceptability, a three-step measurement purification by EFA, PCFA, and CFA, validated and reliable constructs and, measurement of adherence in three distinct domains.

Out-of-pocket expenditure is a major contributor to CRNA which is a prevalent socio-economic problem for Pakistani patients (Hussain et al., 2014; Rizvi et al., 2015; Naqvi et al., 2016, 2018b). Other psychometric tools available are either predominantly formulated to measure patient medication adherence in developed countries or do not account for adherence affected by socio-economic pressure (Forbes et al., 2018; Naqvi and Hassali, 2018). Thus, applicability of other tools in a developing country like Pakistan where most patients pay direct medical cost, is uncertain (Hussain et al., 2014; Naqvi et al., 2016).

The GMAS has incorporated this aspect of adherence and with satisfactory validation results and is deemed a validated tool to measure adherence among patients in developing countries. The validation of GMAS in specific chronic diseases is required. Besides, English translation and validation is a necessary pre-requisite for its international application.

## Conclusion

A novel medication adherence tool known as GMAS was developed in Urdu language. The measurement property of the tool was established. It was deemed validated in Pakistani patients with chronic illnesses.

## Author Contributions

AN and MH conceived the study. AN, MH, MR, AZ, and WI designed the methodology and questionnaire. MR, AZ, and WI obtained ethical approval, conducted data collection and assisted AN and MH in data entry. AN and MH analyzed the data with assistance from AH and SJ. AN wrote the abstract and introduction section with MR. The methodology section was written by AN, MR, AZ, and WI. The result section was written by AN with assistance from AH and SJ. MR, AZ, and WI assisted AN in discussing the findings and drawing conclusions of the study. AH and SJ also contributed in the statistical analysis, validation of the questionnaire and responding to reviewer’s comments. All authors read and approved the final manuscript.

## Conflict of Interest Statement

The authors declare that the research was conducted in the absence of any commercial or financial relationships that could be construed as a potential conflict of interest.

## Supplementary Material

FIGURE S1 GMAS Urdu with scoring code.
