# Supplementary material for: Development and Validation of a Novel General Medication Adherence Scale (GMAS) for Chronic Illness Patients in Pakistan
Source: Front Pharmacol. 2018 Oct 9;9:1124. doi: 10.3389/fphar.2018.01124 (PMC6189444; doi:10.3389/fphar.2018.01124)
Supplement: FIGURE S1 — GMAS Urdu with scoring code. [file Data_Sheet_1.pdf]

Patient # ____________________

ادویات کے استعمال کی جانچ کا سوال نامہ	
درج ذیل سوالات کے لئے کسی ایک جواب کی نشاندہی کریں جو آپ کے دوا لینے کی عادت کے مطابق ہو۔	
مریض کے رویّوں کی وجہ سے (دانستہ و غیر دانستہ) دوا کا ناغہ(GMAS 1)	
1. کیا آپ کو دوائوں کے باقاعدہ استعمال کو یاد رکھنے میں مشکل پیش آتی ہے؟	○ ہمیشہ<br>○ زیادہ تر<br>○ کبھی کبھی<br>○ کبھی نہیں
2. کیا آپ کبھی مصروفیات مثلاً گھریلو یا کاروباری مصروفیات، سفر، ملنا جلنا، پارٹی، شادی بیاہ، مذہبی تہوار کی وجہ سے اپنی دوائیں لینا بھول جاتے ہیں؟	○ ہمیشہ<br>○ زیادہ تر<br>○ کبھی کبھی<br>○ کبھی نہیں
3. کیا آپ خود کو بہتر محسوس کرنے پر ان دوائوں کا استعمال چھوڑ دیتے ہیں؟	○ ہمیشہ<br>○ زیادہ تر<br>○ کبھی کبھی<br>○ کبھی نہیں
4. کیا اس وجہ سے آپ دوائوں کا استعمال چھوڑ دیتے ہیں کہ ان کے منفی اثرات مرتب ہورہے ہیں مثلاً(معدے کی جلن یا نظام ہاضمہ کی خرابی وغیرہ)؟	○ ہمیشہ<br>○ زیادہ تر<br>○ کبھی کبھی<br>○ کبھی نہیں
5. کیا آپ ڈاکٹر کو بتائے بغیر اپنی دوا لینا چھوڑ دیتے ہیں؟	○ ہمیشہ<br>○ زیادہ تر<br>○ کبھی کبھی<br>○ کبھی نہیں
ایک سے زائد بیماریاں یا اضافی دوائوں کا بوجھ (GMAS 2)	
6. کیا آپ اپنی اس بیماری کی دوا کا استعمال اس وجہ سے چھوڑ دیتے ہیں کہ آپ کو اپنی دیگر بیماریوں کی دوائیں کھانی پڑتی ہیں؟	○ ہمیشہ<br>○ زیادہ تر<br>○ کبھی کبھی<br>○ کبھی نہیں
7. کیا نسخے کی پیچیدگی کے سبب آپ کو دوائیں یاد رکھنا مشکل لگتا ہے مثلاً (کھانے سے پہلے یا کھانے کے بعد، آدھی یا پوری گولی کا لینا وغیرہ وغیرہ؟	○ ہمیشہ<br>○ زیادہ تر<br>○ کبھی کبھی<br>○ کبھی نہیں
8. پچھلے مہینے میں کبھی ایسا ہوا ہے کہ آپ اپنے مرض کے بگڑنے کی وجہ سے ، یا اضافی ادویات تجویز ہونے کے سبب اپنی دوا لینا بھول گئے ہوں؟	○ ہمیشہ<br>○ زیادہ تر<br>○ کبھی کبھی<br>○ کبھی نہیں

**9. کیا آپ ڈاکٹر کی تجویز کردہ ادویات کی ترتیب و خوراک ، یا اس کے استعمال میں خود سے ردوبدل کرتے ہیں؟**	○ ہمیشہ<br>○ زیادہ تر<br>○ کبھی کبھی<br>○ کبھی نہیں
**معاشی مسائل کے سبب ناغہ (GMAS 3)**	
**10. کیا دوائیں مہنگی ہونے کی وجہ سے آپ انہیں خرید نہیں پاتے؟**	○ ہمیشہ<br>○ زیادہ تر<br>○ کبھی کبھی<br>○ کبھی نہیں
**11. کیا آپ دوا کا استعمال اس لئے چھوڑ دیتے ہیں کیونکہ یہ ادویات اپنی قیمت کے مطابق فائدہ مند نہیں ہیں؟**	○ ہمیشہ<br>○ زیادہ تر<br>○ کبھی کبھی<br>○ کبھی نہیں

Scoring code

**Grading within domain**<br>High adherence = 13 – 15<br>Good adherence = 11 – 12<br>Partial adherence = 8 – 10<br>Low adherence = 5 – 7<br>Poor adherence = 0 – 4	0<br>1<br>2<br>3	○ ہمیشہ<br>○ زیادہ تر<br>○ کبھی کبھی<br>○ کبھی نہیں	**1. کیا آپ کو دوائوں کے باقاعدہ استعمال کو یاد رکھنے میں مشکل پیش آتی ہے؟**
	0<br>1<br>2<br>3	○ ہمیشہ<br>○ زیادہ تر<br>○ کبھی کبھی<br>○ کبھی نہیں	**2. کیا آپ کبھی مصروفیات مثلاً گھریلو یا کاروباری مصروفیات، سفر، ملنا جلنا، پارٹی، شادی بیاہ، مذہبی تہوار کی وجہ سے اپنی دوائیں لینا بھول جاتے ہیں؟**
	0<br>1<br>2<br>3	○ ہمیشہ<br>○ زیادہ تر<br>○ کبھی کبھی<br>○ کبھی نہیں	**3. کیا آپ خود کو بہتر محسوس کرنے پر ان دوائوں کا استعمال چھوڑ دیتے ہیں؟**
	0<br>1<br>2<br>3	○ ہمیشہ<br>○ زیادہ تر<br>○ کبھی کبھی<br>○ کبھی نہیں	**4. کیا اس وجہ سے آپ دوائوں کا استعمال چھوڑ دیتے ہیں کہ ان کے منفی اثرات مرتب ہورہے ہیں مثلاً(معدے کی جلن یا نظام ہاضمہ کی خرابی وغیرہ)؟**
	0<br>1<br>2<br>3	○ ہمیشہ<br>○ زیادہ تر<br>○ کبھی کبھی<br>○ کبھی نہیں	**5. کیا آپ ڈاکٹر کو بتائے بغیر اپنی دوا لینا چھوڑ دیتے ہیں؟**
		**ایک سے زائد بیماریاں یا اضافی دوائوں کا بوجھ (GMAS 2)**	
**Grading within domain**<br>High adherence = 11 – 12<br>Good adherence = 9 – 10<br>Partial adherence = 6 – 8<br>Low adherence = 4 – 5<br>Poor adherence = 0 – 3	0<br>1<br>2<br>3	○ ہمیشہ<br>○ زیادہ تر<br>○ کبھی کبھی<br>○ کبھی نہیں	**6. کیا آپ اپنی اس بیماری کی دوا کا استعمال اس وجہ سے چھوڑ دیتے ہیں کہ آپ کو اپنی دیگر بیماریوں کی دوائیں کھانی پڑتی ہیں؟**
	0<br>1<br>2<br>3	○ ہمیشہ<br>○ زیادہ تر<br>○ کبھی کبھی<br>○ کبھی نہیں	**7. کیا نسخے کی پیچیدگی کے سبب آپ کو دوائیں یاد رکھنا مشکل لگتا ہے مثلاً (کھانے سے پہلے یا کھانے کے بعد، آدھی یا پوری گولی کا لینا وغیرہ وغیرہ؟**
	0<br>1<br>2<br>3	○ ہمیشہ<br>○ زیادہ تر<br>○ کبھی کبھی<br>○ کبھی نہیں	**8. پچھلے مہینے میں کبھی ایسا ہوا ہے کہ آپ اپنے مرض کے بگڑنے کی وجہ سے ، یا اضافی ادویات تجویز ہونے کے سبب اپنی دوا لینا بھول گئے ہوں؟**
	0<br>1<br>2<br>3	○ ہمیشہ<br>○ زیادہ تر<br>○ کبھی کبھی<br>○ کبھی نہیں	**9. کیا آپ ڈاکٹر کی تجویز کردہ ادویات کی ترتیب و خوراک ، یا اس کے استعمال میں خود سے ردوبدل کرتے ہیں؟**

		**معاشی مسائل کے سبب ناغہ (GMAS 3)**	
**Grading within domain**<br>High adherence = 6<br>Good adherence = 5<br>Partial adherence = 3 – 4<br>Low adherence = 2<br>Poor adherence = 0 – 1	0<br>1<br>2<br>3	○ ہمیشہ<br>○ زیادہ تر<br>○ کبھی کبھی<br>○ کبھی نہیں	**10. کیا دوائیں مہنگی ہونے کی وجہ سے آپ انہیں خرید نہیں پاتے؟**
	0<br>1<br>2<br>3	○ ہمیشہ<br>○ زیادہ تر<br>○ کبھی کبھی<br>○ کبھی نہیں	**11. کیا آپ دوا کا استعمال اس لئے چھوڑ دیتے ہیں کیونکہ یہ ادویات اپنی قیمت کے مطابق فائدہ مند نہیں ہیں؟**
**Grading for overall medication adherence (cumulative)**<br>High Adherence = 30 – 33<br>Good adherence = 27 - 29<br>Partial Adherence = 17 – 26<br>Low Adherence = 11 – 16<br>Poor Adherence = 0 – 10			
